# Preventing Peritoneal Dialysis-Associated Fibrosis by Therapeutic Blunting of Peritoneal Toll-Like Receptor Activity

**DOI:** 10.3389/fphys.2018.01692

**Published:** 2018-11-27

**Authors:** Anne-Catherine Raby, Mario O. Labéta

**Affiliations:** The Wales Kidney Research Unit, Division of Infection and Immunity, School of Medicine, Cardiff University, Cardiff, United Kingdom

**Keywords:** peritoneal dialysis, inflammation, peritoneal fibrosis, toll-like receptors, soluble toll-like receptor 2

## Abstract

Peritoneal dialysis (PD) is an essential daily life-saving treatment for end-stage renal failure. PD therapy is limited by peritoneal inflammation, which leads to peritoneal membrane failure as a result of progressive fibrosis. Peritoneal infections, with the concomitant acute inflammatory response and membrane fibrosis development, worsen PD patient outcomes. Patients who remain infection-free, however, also show evidence of inflammation-induced membrane damage and fibrosis, leading to PD cessation. In this case, uraemia, prolonged exposure to bio-incompatible PD solutions and surgical catheter insertion have been reported to induce sterile peritoneal inflammation and fibrosis as a result of cellular stress or tissue injury. Attempts to reduce inflammation (either infection-induced or sterile) and, thus, minimize fibrosis development in PD have been hampered because the immunological mechanisms underlying this PD-associated pathology remain to be fully defined. Toll-like receptors (TLRs) are central to mediating inflammatory responses by recognizing a wide variety of microorganisms and endogenous components released following cellular stress or generated as a consequence of extracellular matrix degradation during tissue injury. Given the close link between inflammation and fibrosis, recent investigations have evaluated the role that TLRs play in infection-induced and sterile peritoneal fibrosis development during PD. Here, we review the findings and discuss the potential of reducing peritoneal TLR activity by using a TLR inhibitor, soluble TLR2, as a therapeutic strategy to prevent PD-associated peritoneal fibrosis.

## Introduction

Peritoneal dialysis (PD), an essential therapy for end-stage kidney disease, depends on the integrity of the peritoneal membrane. Despite advantages over other dialysis techniques, PD failure due to peritoneal membrane damage remains the major limiting factor (Davies et al., 1999; Williams et al., 2003; Cho et al., 2014). Damage is driven by local peritoneal inflammation, which results in structural alterations of the peritoneal membrane, typically fibrosis – thickening of the sub-mesothelial compact zone – and vascular damage. This leads to altered solute transport through the membrane and dialysis failure (Lambie et al., 2013; Fielding et al., 2014).

Peritoneal infections and the concomitant inflammation resulting from the activity of pathogen-associated molecular patterns (PAMPS) derived from microbial components, are believed to be responsible for 20–40% of PD failure (Cho et al., 2014; Pajek et al., 2014). However, peritoneal inflammation and fibrosis are also observed in PD patients without defined infectious episodes (Tomino, 2012; Cho et al., 2014). In this case, uraemia, prolonged exposure to bio-incompatible PD fluids and surgical catheter insertion have all been reported to induce sterile peritoneal inflammation, fibrosis and membrane failure by promoting tissue damage and cellular stress. This leads to the release and/or generation of endogenous cellular components and matrix degradation products, acting as damage-associated molecular patterns (DAMPs). The DAMPs trigger pro-inflammatory and pro-fibrotic responses (Anders and Schaefer, 2014) that result in local angiogenesis, vasculopathy, epithelial-to-mesenchymal transition in mesothelial cells and collagen deposition in the sub-mesothelial compact zone (Flessner et al., 2007; Johnson et al., 2012; Tomino, 2012; Cho et al., 2014; Strippoli et al., 2016).

The immune mechanisms linking infection-induced or sterile inflammation with the onset, development and regulation of PD-associated peritoneal fibrosis are poorly defined and thus the focus of intense investigation (Fielding et al., 2014; Liappas et al., 2015, 2016; Raby et al., 2018). Consequently, effective therapies to prevent PD-associated fibrosis remain to be developed.

Critical to triggering pro-inflammatory responses is the activity of the Toll-like family of innate immune receptors (TLRs) (Kawai and Akira, 2010; Kawasaki and Kawai, 2014). TLRs are expressed in a variety of cell types, including peritoneal leukocytes and mesothelial cells (Colmont et al., 2011; Raby et al., 2017). They recognize a wide range of microorganisms and their PAMPs (e.g., lipopolysaccharide/endotoxin/LPS; lipopeptides) as well as DAMPs released as a consequence of cellular stress [e.g., High Mobility Group Box-1 (HMGB-1); heat shock proteins (Hsp)], or generated following extracellullar matrix degradation during tissue damage (e.g., hyaluronan, fibronectin) (Chen and Nuñez, 2010; Anders and Schaefer, 2014). TLR triggering results in the production of potent pro-inflammatory and fibrotic mediators, e.g., IL-6, TGF-β, TNF-α, IL-8, IFN-γ, and IL-1β (Fielding et al., 2014; Kawasaki and Kawai, 2014).

Inappropriate TLR activation may result in serious inflammatory conditions, therefore, they are being considered as therapeutic targets for the prevention and/or treatment of a number of inflammatory pathologies (Riedemann et al., 2003; Kanzler et al., 2007; Mollnes et al., 2008; Dunne et al., 2011; Raby et al., 2013). Given the close link between inflammation and fibrosis, and the recognized involvement of TLRs in tissue fibrosis (Anders and Schaefer, 2014), we recently assessed the role that TLRs play in peritoneal fibrosis development during PD (Raby et al., 2017, 2018). Here, we review the findings and discuss the potential of reducing peritoneal TLR activity by using the soluble form of TLR2, a TLR modulator, as a therapeutic strategy to prevent PD-associated peritoneal fibrosis.

## Critical Contributions of TLR2 and TLR4 to Pd-Associated Peritoneal Macrophage and Mesothelial Cell Pro-Inflammatory and Fibrotic Responses

### TLR2- and TLR4-Mediated Peritoneal Macrophage and Mesothelial Cell Responses to Infection

Recent studies have focused on TLR2 and TLR4, as these TLRs recognize the widest range of microbial components involved in PD-associated infections and are also the main TLRs involved in sterile inflammatory responses (Anders and Schaefer, 2014; Kawasaki and Kawai, 2014).

Consistent with their expression detected in PD effluent (PDE)-isolated uremic leukocytes, TLR2 and TLR4 were found to mediate pro-inflammatory (IL-6, IL-8, and TNF-α) and fibrotic (TGF-β, IL-6, IL-13, MMP1, MMP3, MMP9, and TIMP-1) responses in PDE leukocytes stimulated with the Gram-positive bacterium *Staphylococcus epidermidis*, Pam_3_-Cys-Ser-(Lys)_4_ (Pam_3_Cys, a synthetic bacterial lipopeptide) – both TLR2 agonists – the Gram-negative bacterium *Escherichia coli* and the Gram-negative bacterial cell-wall component LPS – both TLR4 agonists. Macrophages were the main cell type responsible for the observed leukocyte responses, consistent with their high TLR receptor expression compared with lymphocytes (Raby et al., 2017).

Similar to peritoneal leukocytes, human peritoneal mesothelial cells (HPMC, from greater omentum) were found to respond to Pam_3_Cys, *S. epidermidis* and *E. coli*, but not to LPS. HPMC’s lack of response to LPS reflected the documented lack of TLR4 expression in HPMC (Colmont et al., 2011). However, HPMC responded to *E. coli*, most likely by recognizing bacterial lipopeptides through TLR2 and flagellin – the protein component of the flagellum of Gram-negative bacteria – through TLR5 expressed in these cells (Colmont et al., 2011).

**FIGURE 1:**
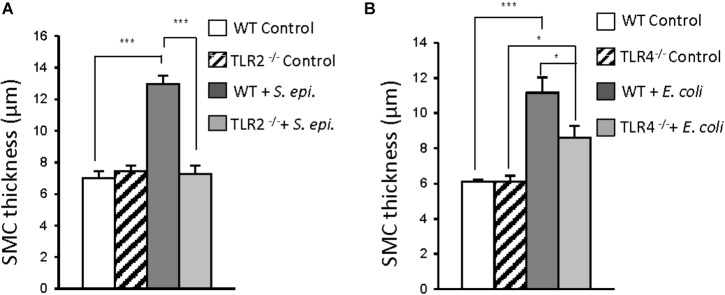
Critical contribution of TLR2 and TLR4 to bacteria-induced peritoneal fibrosis development. **(A,B)** Wild-type (WT), TLR2 deficient (TLR2^-/-^) or TLR4^-/-^ mice (*n* = 5 per group) were inoculated intraperitoneally 4 times at weekly intervals with *S. epidermidis* (*S. epi*., 5 × 10^8^ CFU/mouse) or *Escherichia coli* (*E. coli*, 2 × 10^7^ CFU/mouse) or left untreated (control). Four weeks after the last injection, histological analysis of the peritoneal membrane was conducted and the thickness of the sub-mesothelial compact zone (SMC, layer between the muscle and membrane surface) was determined. Bar plots show the mean ( ± SEM) of SMC thickness in each experimental group. ^∗^*P* < 0.05; ^∗∗∗^, *P* < 0.005. Adapted with permission from Raby et al. (2017).

*In vivo* studies confirmed the critical role that TLR2 and TLR4 play in infection-induced peritoneal inflammation and fibrosis (Raby et al., 2017). A mouse model of peritoneal inflammation and fibrosis induced by repeated intraperitoneal injections of *S. epidermidis* (TLR2 agonist) or *E. coli* (TLR4 agonist) was used. This model mimics the typical clinical episodes of recurrent bacterial peritonitis leading to peritoneal fibrosis observed in PD patients (Fielding et al., 2014). Repeated injection of *S. epidermidis* in wild-type (WT) mice resulted in substantial peritoneal fibrosis, whereas *S. epidermidis* injection in TLR2-deficient mice did not result in fibrosis development (Figure 1A). By contrast, injection of *E. coli* in TLR4-deficient mice resulted in a partial reduction in fibrosis when compared with WT mice (Figure 1B). This is consistent with the possibility that *E. coli*-induced pro-fibrotic responses may involve other receptors (e.g., TLR2, TLR5) in addition to TLR4. Together, these findings indicated a major role for TLR2 and to a lesser extent for TLR4 in bacteria-induced peritoneal fibrosis associated with PD, and pointed at controlling infection-induced TLR-mediated activation as a potential therapeutic against peritoneal fibrosis.

### TLR2- and TLR4-Mediated Peritoneal Macrophage and Mesothelial Cell Responses to PD Solutions

The role of TLR2 and TLR4 in sterile inflammatory and fibrotic responses of peritoneal cells resulting from exposure to PD solutions (PDS) was also evaluated (Raby et al., 2018). A number of PDS elicited pro-inflammatory and pro-fibrotic responses (CXCL-8/IL-8, IL-6, TNF-α, TGF-β, and IL-1β) from PDE-isolated uremic peritoneal leukocytes and mesothelial cells (from greater omentum), including those glucose-based (1.36 and 2.27% glucose Dianeal^®^, Physioneal^®^, Stay Safe^®^) or icodextrin-based (Extraneal^®^), having low pH (Dianeal^®^, Extraneal^®^, Stay Safe^®^) or physiologic pH (Physioneal^®^).

Interestingly, analysis of the expression of inflammatory and immunity-related genes in uremic peritoneal leukocytes and HPMC exposed from 16 h to low glucose Dianeal^®^ (1.36% glucose), a commonly used PDS, showed substantial modulation of a number of genes. In leukocytes, 15 genes were found significantly up-regulated by Dianeal^®^, and only 5 were down-modulated. The transcripts up-modulated by PDS included those coding for inflammatory mediators (CXCL8/IL-8, TNF-α, IFN-γ, monocyte chemoattractant CCL2/MCP-1, the chemokine receptor CCR4, IL-1β) as well as for TLR2, TLR1, and TLR6 (TLR2 signaling partners), TLR3 and TLR signal intermediates.

In HPMC, 8 genes were found up-regulated and 6 down-regulated following exposure to Dianeal^®^. The transcripts for the pro-inflammatory cytokines IL-1α, IL-1β, and CXCL8/IL-8 were strongly up-modulated, whereas that for CXCL10/IL-10 – an anti-inflammatory cytokine – was found down-modulated. Fibrosis-related gene expression analysis in Dianeal^®^-exposed HPMC – the cell type that contributes to peritoneal fibrosis by acquiring a fibroblastic phenotype following epithelial-to-mesenchymal transdifferentiation (EMT) – showed a 3-fold increase in *VGEFA* (main isoform of VGEF) expression and a reduction in *E-cadherin*, both effects indicating EMT (Yung and Chan, 2012; Ruiz-Carpio et al., 2017).

Notably, peritoneal leukocyte TLR2 or TLR4 blocking with specific monoclonal antibodies inhibited the pro-inflammatory cytokine release induced by Dianeal^®^, and the extent of the inhibition depended on the PD patient tested. Simultaneous blocking of TLR2 and TLR4 resulted in a stronger inhibition of a number of pro-inflammatory and fibrotic cytokines released by the PDS-exposed uremic peritoneal leukocytes. TLR2 blockade in PDS-exposed HPMC also showed a significant reduction in pro-inflammatory mediator release. Together, these findings indicated that peritoneal TLR2 and TLR4 control inflammatory and fibrotic responses to PDS exposure.

Interestingly, it was found that the cellular stress resulting from PDS exposure induces DAMP generation which in turn triggers TLR2 and TLR4 activation, and that the PDS does not contain pre-existing components capable of TLR activation. Of note, Hsp70 and low (∼33 kDa) and medium (∼289 kDa) molecular mass hyaluronan (HA) were identified as the main PDS-induced DAMPs. They elicited inflammatory responses from peritoneal cells through TLR2/TLR4 activation, as Hsp70 and HA are ligands of both TLR2 and TLR4 and their specific inhibition reduced PDS-induced inflammation in peritoneal leukocytes.

It is worth noting that, in addition to eliciting inflammatory responses, heat-shock proteins have shown cytoprotective activity against cytotoxicity resulting from PDS exposure (Kratochwill et al., 2009). It is believed that peritoneal damage due to PD exposure may reflect an imbalance between cellular injury-induced inflammation and cytoprotective processes. The extracellular exposure to otherwise intracellular cytoprotective molecules such as Hsp70, released as a consequence of tissue damage/cell death, may trigger DAMP signals leading to pro-inflammatory responses and exacerbating peritoneal damage (Kratochwill et al., 2011).

These findings suggested that inhibiting DAMP-TLR associations may have therapeutic potential against peritoneal fibrosis induced by PDS exposure.

## Therapeutic Potential of Soluble TLR2 Against Infection-Induced and Sterile Peritoneal Inflammation and Fibrosis Associated With PD

The therapeutic potential of inhibiting infection- or PDS-induced TLR activation to prevent peritoneal fibrosis development was evaluated by testing the ability of soluble Toll-like receptor 2 (sTLR2), a TLR inhibitor, to regulate peritoneal inflammation. It is well documented that sTLR2 reduces TLR-mediated inflammation by both acting as a decoy receptor, binding to TLR2 ligands, and by interfering with the co-receptor activity of CD14, the main co-receptor for most TLRs (Lebouder et al., 2003; Raby et al., 2009, 2013).

### Inhibitory Effect of sTLR2 on PD-Associated Peritoneal Infection-Induced Inflammation and Fibrosis

**FIGURE 2:**
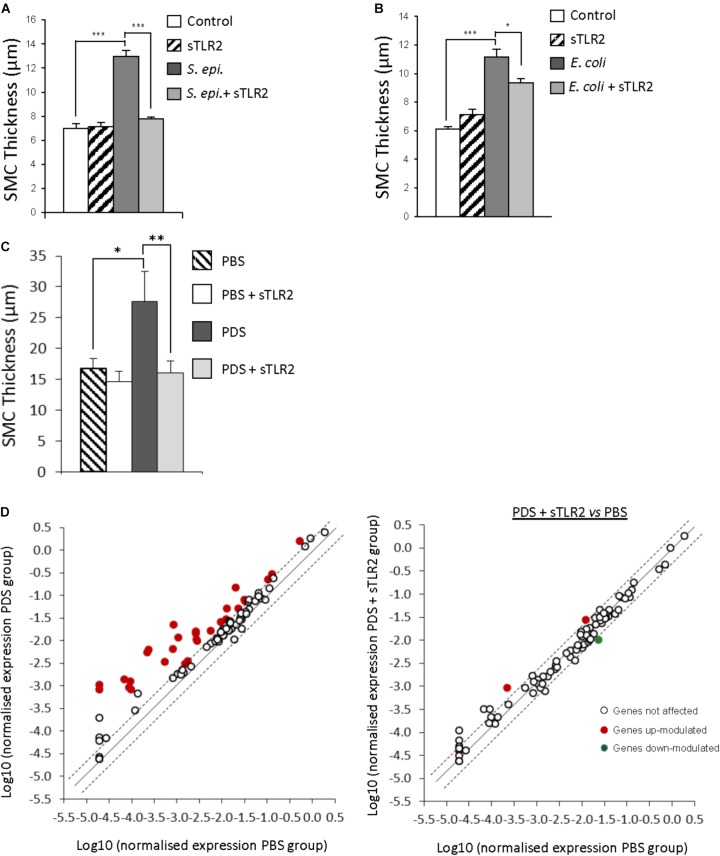
Therapeutic potential of soluble Toll-like receptor 2 (sTLR2) against bacteria- and PD solution-induced peritoneal fibrosis development. **(A,B)** mice (*n* = 5 per group) were inoculated intraperitoneally 4 times at weekly intervals with *S. epidermidis* (*S. epi*., 5 × 10^8^ CFU/mouse) or *Escherichia coli* (*E. coli*, 2 × 10^7^ CFU/mouse) in the presence or absence of sTLR2 (250 ng/mouse), or left untreated (control). Four weeks after the last injection, histological analysis of the peritoneal membrane was conducted and the thickness of the sub-mesothelial compact zone (SMC) was determined. Bar plots show the mean ( ± SEM) of SMC thickness in each experimental group. ^∗^ *P* < 0.05; ^∗∗∗^, *P* < 0.005. **(C,D)** Mice were instilled twice daily with 2 ml of PBS (*n* = 5) or Fresenius Standard glucose solution (PDS, *n* = 8) in the presence or absence of sTLR2 for 40 days before sacrifice, tissue sample collection and histological analysis of the peritoneal membrane for SMC thickness determination. Results show the mean ( ± SEM) for each experimental group. ^∗^*P* < 0.05; ^∗∗^*P* < 0.01. Scatter plots in **(D)** show the effect of PDS on the expression of fibrosis-related genes in the absence and presence of sTLR2, as assessed by quantitative RT-PCR on RNA extracted from peritoneal membrane samples. Dotted lines indicate the 0.5 and 2 fold change thresholds. Open circles outside the dotted lines correspond to genes modulated in a non-statistically significant manner. Adapted with permission from Raby et al. (2018).

When administered together with the repeated peritoneal injection of *S. epidermidis* in mice, sTLR2, was found to prevent fibrosis development (Figure 2A; Raby et al., 2017). This effect was accompanied by a substantial reduction of inflammatory parameters, including the peritoneal levels of a number of pro-inflammatory cytokines and chemokines, neutrophils (PMN) and monocytes at the peak time of their influx to the peritoneum as well as the prototypical pro-fibrotic cytokine TGF-β. Of note, in spite of reducing inflammation and phagocyte recruitment, the capacity of the mice to clear the infection was not found affected by the presence of sTLR2, as no difference in bacterial load (peritoneum and blood) between mice treated and non-treated with sTLR2 was observed.

Fibrosis-related gene transcripts were also markedly inhibited by sTLR2 administration. Of the 85 genes tested, 21 were found markedly up-regulated by *S. epidermidis*, and sTLR2 reduced this effect in 18 of them. The transcripts reduced by sTLR2 included *Fasl*, central to apoptosis, which impairs bacterial clearance during PD (Hohlbaum et al., 2001; Catalan et al., 2003); *STAT-1*, a critical signal intermediate for fibrosis development (Fielding et al., 2014), and *IL-6* – a major promoter of peritoneal fibrosis (Fielding et al., 2014). Notably, sTLR2 counteracted *S. epidermidis’* negative effect on matrix metalloproteinases (MMPs) Mmp-1, Mmp-3 and Mmp-9, and *S. epidermidis*’ positive effect on Mmp-13 and the MMP inhibitor Timp-1 (Raby et al., 2017).

Of note, peritoneal fibrosis induced by Gram-negative bacteria was also inhibited by sTLR2, as simultaneous peritoneal inoculation of sTLR2 with the repeated injection of *E. coli* resulted in reduced peritoneal fibrosis (Figure 2B). This reflects the fact that, in spite of not acting as a TLR decoy receptor for most Gram-negative bacterial components, sTLR2 can still reduce TLR-mediated fibrotic signaling induced by Gram-negative bacteria by inhibiting CD14, a co-receptor for most TLRs (Raby et al., 2009), including TLR4. Thus, peritoneal fibrosis resulting from repeated peritoneal bacterial infections like those associated with PD can be inhibited by sTLR2 by acting on a variety of pro-inflammatory and fibrotic mediators, but notably, without affecting infection clearance.

### Inhibitory Effect of sTLR2 on PDS-Induced Peritoneal Inflammation and Fibrosis

The therapeutic potential of sTLR2 against inflammation and fibrosis development resulting from prolonged peritoneal exposure to PDS was tested in a murine model of sterile peritoneal fibrosis consisting of daily peritoneal catheter infusions of a standard PDS (Raby et al., 2018). This mouse model mimics the changes in the peritoneal membrane (morphological and functional) observed in non-infected patients on PD (Gonzalez-Mateo et al., 2009; Loureiro et al., 2011). The peritoneal administration of sTLR2 together with the PDS twice weekly prevented the development of peritoneal fibrosis (Figure 2C). In agreement with this finding, sTLR2 was found to suppress the PDS-induced increased expression of inflammatory and fibrotic mediators (TNF-α, IL-1β, KC, IL-6, and IFN-γ). The suppressive effect of sTLR2 on inflammatory mediators correlated with a substantial reduction in the number of peritoneal leukocytes and the percentage of infiltrating neutrophils in particular (Raby et al., 2018). Notably, sTLR2 counteracted the negative effect of PDS on regulatory T cell (Treg) numbers, recovering their numbers to the levels observed following PBS inoculation. Tregs, an anti-inflammatory T cell subset, control T cell expansion, including that of Th17 cells, an inflammatory T cell subset involved in peritoneal damage and fibrosis development (Liappas et al., 2016). sTLR2’s positive effect on Treg cells resulted in an increased in the Treg:Th17 ratio.

Analysis of fibrosis-related gene transcripts in mice peritoneal membranes carried out after the last inoculation of PDS+sTLR2 showed that sTLR2 also counteracted the positive effect of PDS on mRNA coding for several inflammatory mediators and fibrosis markers (Figure 2D). Of the 85 genes tested, 29 were markedly up-regulated by PDS at this time point, and sTLR2 was found to reduce this effect in 27 of them, including in the transcripts for FasL, STAT-1, IFN-γ, MMPs, TIMP1/3, TGF-β, IL-1β, and TNF-α. Thus, the development of peritoneal fibrosis by long exposure to PDS can be prevented by administering sTLR2, which inhibits pro-inflammatory and fibrotic mediator production and controls the expansion of inflammatory cells.

## Conclusion

The results of recent investigations reviewed here revealed the critical role that peritoneal TLR2 and TLR4, main members of the Toll-like family of innate immune receptors, play in mediating inflammation and fibrosis induced either by recurrent peritoneal infections during PD or prolonged exposure to PD solutions. Furthermore, the investigations showed the potential of a novel therapeutic strategy that targets TLRs to blunt peritoneal inflammation and thus prevent fibrosis development (either infection-induced or sterile) during PD by the use of a decoy receptor, sTLR2. This soluble receptor also inhibits the activity of CD14, the common TLR co-receptor. Thus, sTLR2 can reduce pro-inflammatory and fibrotic responses to different pathogens (e.g., Gram-positive and Gram-negative bacteria) and their PAMPs and to endogenous TLR ligands (DAMPs) activating different TLRs, not only TLR2. These findings pave the way for future clinical trials to test the clinical efficacy of sTLR2 as a therapy for patients in long-term PD.

Notably, the preclinical studies showed that peritoneal inflammation and fibrosis induced by bacteria in mice can be inhibited by sTLR2 without affecting the animal’s capacity to resolve the infection. Given that PD patients are prone to infections, this ability of sTLR2 would be advantageous when comparing with complete TLR blockade-based therapies, e.g., by combination of anti-TLR2 and -TLR4 antibodies (Lima et al., 2015), as these may have a detrimental effect on infection clearance. However, preclinical studies have shown the potential of combining anti-TLR2 and TLR4 antibodies with antibiotics to reduce inflammation whilst controlling infection (Spiller et al., 2008; Lima et al., 2015). Thus, a comparative evaluation of the efficacy of both TLR-targeting therapeutic strategies in PD models of infection/fibrosis will be required. Similarly, the efficacy of sTLR2 as a treatment for established fibrosis and membrane failure remains to be evaluated, since in the reported studies sTLR2 was inoculated together with the infecting bacteria or the PD solution in an initially healthy peritoneal membrane.

The pro-fibrotic cytokine TGF-β has been a main target for therapeutic interventions. Inhibition of its synthesis or activity showed promising effects (Duman et al., 2001; Margetts et al., 2002; Kyuden et al., 2005; Loureiro et al., 2011; Tomino, 2012; Zhang et al., 2014; Nongnuch et al., 2015). However, given TGF-β pleiotropic functions, its blockade is potentially hazardous (Blobe et al., 2000; Yoshimura et al., 2010), and it is just one of several mediators of fibrosis acting down-stream of TLR activation.

Thus, the reported sTLR2-based anti-fibrotic strategy may be a valuable complement to antibiotic therapies during PD infections, to biocompatible PDS or to PDS supplemented with immunomodulatory dipeptides to mitigate the PDS’ adverse effects (Ferrantelli et al., 2016). sTLR2 may also be useful in other inflammatory conditions associated with PD, for example to help reduce the increased risk of cardiovascular diseases.

## Author Contributions

ML proposed the subject and conceived the general structure of the review. A-CR and ML revised the existing literature and contributed to all the sections.

## Conflict of Interest Statement

The authors declare that the research was conducted in the absence of any commercial or financial relationships that could be construed as a potential conflict of interest.
